# Exercise and eating behaviour: the role of mindfulness

**DOI:** 10.1186/2050-2974-1-S1-O24

**Published:** 2013-11-14

**Authors:** Rachel Martin, Ivanka Prichard, Amanda D Hutchinson, Carlene Wilson

**Affiliations:** 1 School of Psychology, Flinders University, Australia; 2 Flinders Centre for Innovation in Cancer, School of Medicine, Australia

## 

The present study examined yoga participation and cardiovascular exercise in relation to dietary intake and disordered eating symptomatology and the role of mindfulness. Participants were 159 female exercisers who completed a questionnaire incorporating measures of exercise behaviour, body awareness, trait mindfulness, mindful eating, and dietary intake and disordered eating symptomatology. Participation in yoga was associated with significantly lower levels of disordered eating symptomatology whereas the amount of time spent participating in cardio-based exercise was associated with greater eating disturbance. The relationship between yoga participation and eating behaviour was mediated by both trait mindfulness and body awareness; the relationship between cardio-based exercise and eating behaviour was partially mediated by trait mindfulness. The relationships between amount of exercise and actual food intake were not mediated by trait mindfulness or body awareness. The differential findings for dietary intake and disordered eating proneness indicate that mindfulness may be more beneficial for clinical populations or those at risk for eating disorders than for modifying actual dietary intake in the general population.

This abstract was presented in the **Disordered Eating – Characteristics & Treatment** stream of the 2013 ANZAED Conference.

